# Dynamically tunable robust ultrahigh-Q merging bound states in the continuum in phase-change materials metasurface

**DOI:** 10.1515/nanoph-2024-0557

**Published:** 2025-01-31

**Authors:** Hui Ren, Jietao Liu, Zengxuan Jiang, Lingyun Zhuang, Botao Jiang, Chunhao Xu, Bo Cheng, Guofeng Song

**Affiliations:** Nano Optoelectronics Laboratory, Institute of Semiconductors, Chinese Academy of Sciences, Beijing 100083, China; College of Materials Science and Opto-Electronic Technology, University of Chinese Academy of Sciences, Beijing 100049, China; Institute of Intelligent Photonics, 12538 Nankai University, Tianjin, China

**Keywords:** merging BICs, phase change materials, tunability

## Abstract

Bound states in the continuum (BICs) are localized states within the radiative continuum that exhibit high quality-factor (Q-factor) resonance, which significantly boosts light–matter interactions. However, out-of-plane radiation losses can arise from inherent material absorption and inevitable technological imperfections during fabrication process. Merging BICs have been introduced as a solution to address the issue of out-of-plane radiation losses. By merging BICs, it is possible to expand the area of high Q-factor resonance in momentum space, thereby enhancing the system’s robustness against external perturbations. However, achieving this enhancement is contingent upon altering the geometrical parameters of the structure, which inherently restricts its dynamic tunability. Here, we propose an emerging approach that integrates phase change materials (PCMs) into photonic crystal slabs (PCs) metasurface, enabling dynamically tuning of merged BICs. By utilizing low-loss Sb_2_S_3_ as a tunable PCMs, we demonstrate that altering its phase state can merge BICs, leading to a substantial increase in the high Q-factor across an extended range of wave vectors space. Furthermore, this study validates the universality and robustness of merging BICs against common unit-cell topology fabrication defects. Additionally, by twisting the square holes to break in-plane symmetry, asymmetric merging and inversion of topological charge at the ***Γ***-point are achieved. This approach leverages phase-transition states of PCMs to enable reconfigurable polarization distribution of radiation field without scale and parameter changes, which is tunable and offers promising potential applications in optical vortices and nano-lasers.

## 1 Introduction

Bound states in the continuum (BICs) are localized states that exist within the continuum alongside extended waves, but they are completely confined without any radiation [1]. Remarkably, BICs have been incorporated into a wide range of metasurfaces and a variety of photonic structures [2], including photonic crystal slabs (PCs) [3], micro-ring resonator [4], photonic waveguides [5], photonic crystal fibers [6]. In additional, the integration of tunable BICs metasurfaces into another tunable material can enhance coupling and absorption. For example, a single layer of MoS_2_ and WS_2_ were integrated into asymmetric dielectric metasurface composed of TiO_2_ meta-atoms with a broken in-plane inversion symmetry on an SiO_2_ substrate, respectively. The high-Q resonance supported by quasi-BICs is realized, with the absorption efficiency significantly enhanced by the synergistic effects of magnetic dipole resonance and exciton coupling [7], [8]. An ideal BICs has an infinite quality factor (Q-factor) [9]. In PCs, in addition to the symmetry-protected BICs [10], which is usually fixed at the ***Γ***-point, there also exists accidental BICs [11]. By exploiting the topological nature of BICs [12], [13], multiple BICs on the same photonic band can be tuned to the same wavevector to create a merging BICs [14]. The merging BICs can suppress out-of-plane scattering losses and reduce the radiation losses of the resonator [15], [16], [17]. This results in achieving a high Q-factor across a broad range of spatial frequencies and reducing structural sensitivity, thus rendering it more resilient to external perturbations. *Jin* et al. demonstrated the numerical results of merging BICs at the ***Γ***-point using PCs with a square lattice pattern of circular holes, where the Q-factor of merging BIC was approximately 10 times higher than that of non-merging BICs [14]. *Meng Kang* et al. conducted numerical verification merging symmetry-protected BICs and accidental BICs at off ***Γ***-point [12], as well as the construction of merging BIC with higher-order BICs, respectively [18].

However, the approaches to merging BICs all entail modifications to structural parameters, such as the period and thickness of the PCs [12], [18], which significantly constrains the tunability and flexibility of merging BICs. Therefore, more flexible methods for constructing merging BICs [19], [20] are desired. To achieve this, we present a solution of integrating PCMs into metasurface. PCMs can undergo significant changes in optical properties during the transition from amorphous to crystalline states, making them an appealing choice for photonic applications [21]. Such phase transitions can be accomplished by high-intensity electrical impulses, and they are non-volatile, fast (at nanoseconds), and reversible [22], [23]. The integration of metasurfaces with the adjustable optical properties of PCMs presents promising prospects for the development of active nanophotonic devices. Barreda et al. combined PCMs and metasurfaces to manipulate BICs through amorphous and crystalline transitions of PCMs to obtain the “on"/"off’ switching effects of quasi-BIC resonances for Ge_2_Sb_2_Te_5_ [24]. While the utilization of PCMs in metasurfaces to dynamically tune quasi-BICs has been extensively studied, their application in manipulating merging BICs remains unexplored.

In this paper, we validate the manipulation of BICs by PCM-metasurfaces, achieving merging BICs with Ultrahigh Q-factors. To this end, we propose a novel PCMs-integrated PCs that exploits the difference in optical properties before and after the PCMs phase transition to achieve the manipulation of BICs in momentum space, which enhances the tunability as compared to that by changing the structural parameters. To obtain high Q value, low-loss Sb_2_S_3_ PCMs [25], [26]is employed. By altering the phase state of Sb_2_S_3_, it was possible to achieve BICs merging and realize higher Q-factor values with slower decay. Specifically, the Q-factor of single isolated BICs decays quadratically $\left(Q\propto k^{-2}\right)$ with respect to the wavevector *k* (in momentum space), while the scaling of the merging BICs becomes *Q* ∝ *k* ^−6^. The theoretical explanation is provided for the effects of PCMs on the merging mechanism. Additionally, numerical verification demonstrates that pore shape does not affect BICs merging. Furthermore, breaking in-plane symmetry of PCs enables asymmetric merging of BICs as well as topological charge reversal, which sheds lights on applications in vortex generators.

## 2 Materials and methods

**Figure 1:**
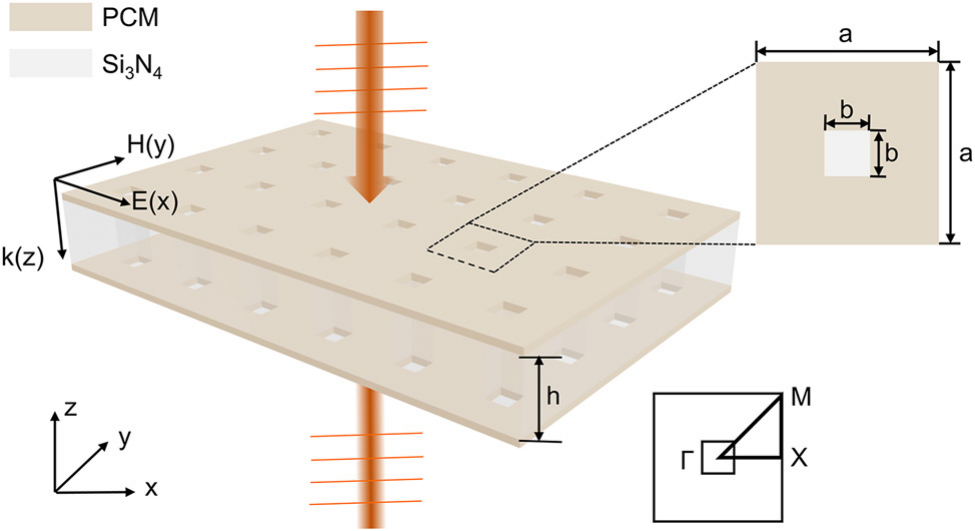
Three-dimensional model and planar schematic of the designed structure. The corner shows the wave vector scanning region of the First Brillouin Zone.

We designed a metasurface of a PCs operating in the near-infrared region, which is a hollow periodic array with C_4v_ symmetry, as shown in Figure 1. The PCs are composed of Si_3_N_4_ (refractive index of n=2.02) sandwiched between upper and lower layers of PCMs (Sb_2_S_3_), with periodic etched square holes. Compared with other PCMs such as Ge_2_Sb_2_Te_5_ [21], Sb_2_S_3_ has low optical losses in the near infrared and even within the visible spectrum [27], which facilitates the novel construction of reconfigurable photonic devices. The optical properties of Sb_2_S_3_ are detailed in Table 1 [28]. More detailed results can be found in the Supplementary information.

**Table 1:** Values of refractive index $\tilde{n}\;=\;n\;+\;ik$ of Sb_2_S_3_ in amorphous and crystalline phases, for selected wavelengths [28].

States	Wavelengths				
	633 nm	800 nm	1060 nm	1310 nm	1550 nm
Amorphous	3.148+0i	2.943+0i	2.829+0i	2.767+0i	2.717+0i
Crystalline	3.989+0.267i	3.630+0.008i	3.413+0i	3.343+0i	3.308+0i

The Figure 1 depicts the unit cell structure, the period is *a* = 600 nm, the width of the etched hole is *b* = 160 nm, the height of the PCs is *h* = 640 nm, and the thickness of the PCMs in both upper and lower layers is 10 nm. To achieve mirror symmetry in the *z*-direction, the domain above and below the metasurface are set as air cladding layers. We have used the finite element method (COMSOL Multiphysics) to calculate the eigenmode field distributions and Q-factor values.

## 3 Results and discussion

### 3.1 Moving of BICs by translating the phase state of PCMs

**Figure 2:**
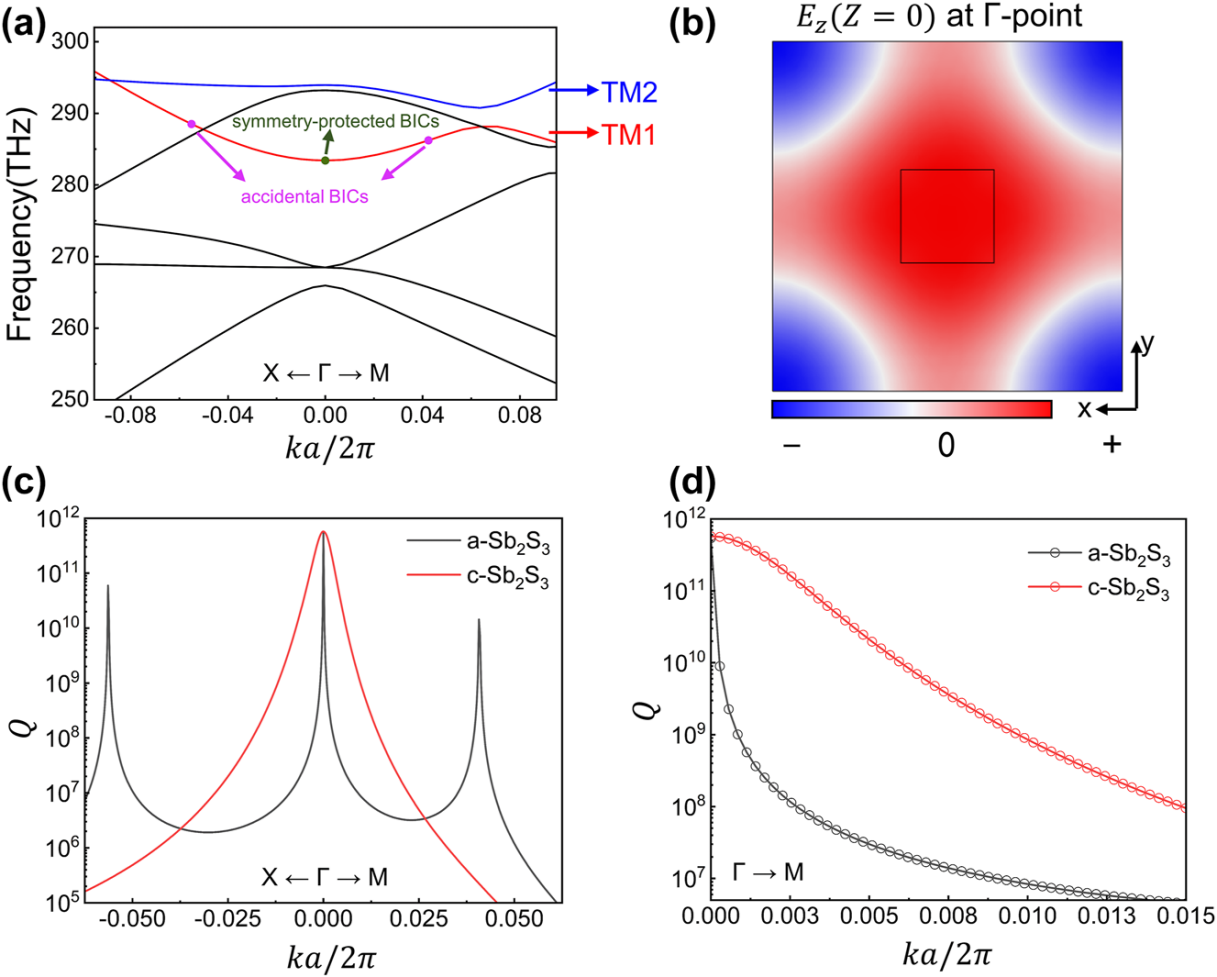
Energy band and Q-factor of the metasurface. (a) Calculated band structure. The specific BICs in energy band is highlighted with dots and arrows. (b) The electric field *z*-component profile (Z=0) for one unit at *Γ*-point for the crystalline state PCMs. (c) Calculated Q-factor values for Sb_2_S_3_ in its amorphous (black) and crystalline (red) states. Upon transformation to the crystalline state (red), a merging BIC is formed, leading to high Q-factor achieved over a wider range of spatial frequencies. (d) The Q-factor scaling rules exhibit changes before (black) and after (red) the merge of the BICs, varying as a function of the wave vector along the ***Γ-M*** direction.

To better understand the underlying physics, we investigate the merging process of PCs by conducting mode analysis. Figure 2(a) depicts the calculated energy bands, where the TM (transverse magnetic) mode of TM1 energy band (red curve) includes symmetry-protected BICs at the ***Γ***-point and accidental BICs at each highly symmetry axis, while the second mode TM2 energy band has only symmetry-protected BICs located at the ***Γ***-point, and thus we focus on the TM1 energy band. The proximity of the accidental BICs to the ***Γ***-point is advantageous for subsequent tuning. Figure 2(b) illustrates the electric field distribution within the band at the ***Γ***-point, highlighting the predominance of the *E*_*z*_ components, which is characteristic of TM-like modes. We have investigated the merging process of this energy band along the ***X-Γ-M*** direction by transforming the state of Sb_2_S_3_ from amorphous to crystalline, corresponding to different curve-colors (Figure 1(c)). When Sb_2_S_3_ is in the crystalline state, the separated accidental BICs located at $k\;=\;\frac{0.03a}{2\pi }$ migrate towards the ***Γ***-point to form a merging BIC. The change in Q-factor before and after merging can be characterized by scaling rules [14], as shown in Figure 2(d). The result illustrates the fitting of Q-factor in two distinct scenarios; it is evident that for the isolated accidental BICs (depicted in black-line with hollow circles), the geometric decay of Q-factor follows $Q\propto \frac{1}{{\left[k\left(k+k_{\text{accidental}}\right)\left(k-k_{\text{accidental}}\right)\right]}^{2}}$ [29]. Upon transition of Sb_2_S_3_ to its crystalline state, the decay of Q-factor for the merging BIC becomes $Q\propto \frac{1}{k^{6}}$ (depicted in red-line with hollow circles), significantly lower than that for isolated accidental BICs.

**Figure 3:**
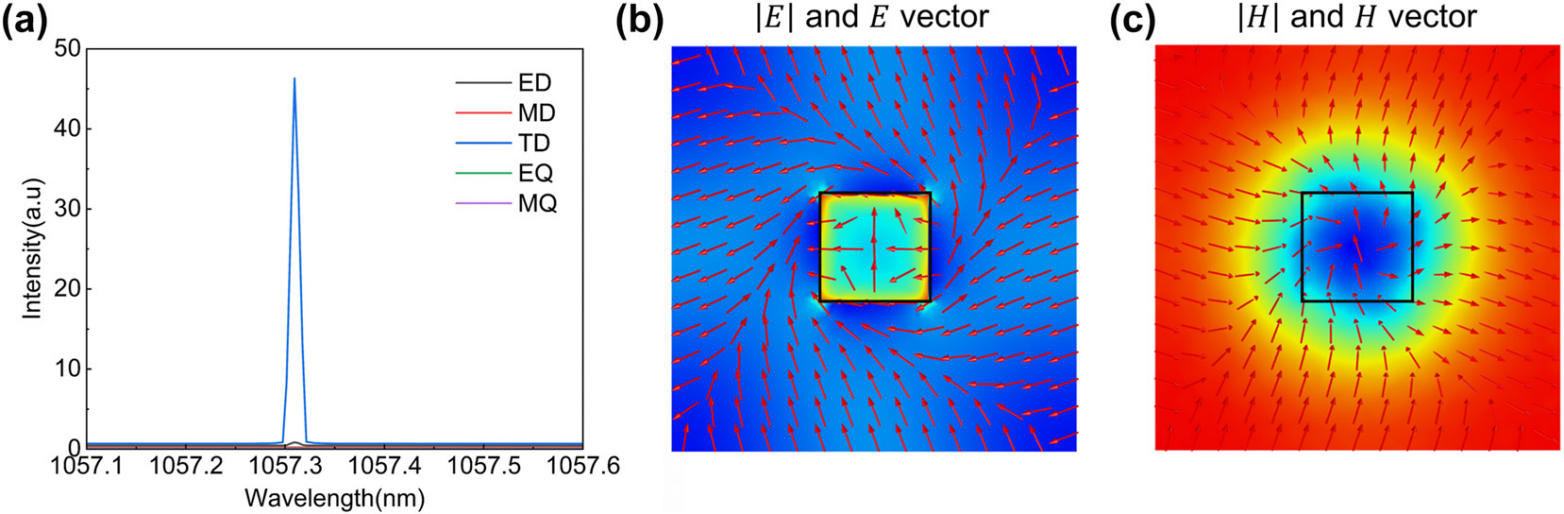
Multipole expansion and mode profile. (a) Multipole decomposition analysis reveals that the selected bands are predominantly influenced by a toroidal dipole mode. The abbreviations ED, MD, TD, EQ and MQ denote electric dipole, magnetic dipole, toroidal dipole, electric quadrupole, and magnetic quadrupole, respectively (b–c) Mode profiles, along with the corresponding electric field vectors (left) and magnetic field vectors (right), demonstrating the presence of the dipole mode in the ***x-y*** cross section.

Based on the multipole decomposition analysis of the Cartesian coordinate system, we have investigated the components of the individual modes of the TM1 band, as shown in Figure 3(a). The toroidal dipole mode is dominant due to its weak coupling to incident light and relatively large Q-factor, which provides better merging of the BICs scaling rules in *k-*space [30]. This can be observed from the results of the electric (*E*) and magnetic (*H*) fields distribution shown in Figure 3(b and c), where the toroidal dipole mode with a circular electric field and corresponding toroidal magnetic field is seen.

The tunability of the resonant mode is attributed to the interface discontinuity of the electric field, elucidating the physical mechanism underlying this phenomenon [31]. The TM-like electromagnetic field can be given by (***H***_***x***_ ***, H***_***y***_ ***, E***_***z***_) that in our case. We can obtain the coupling equations of different channels from Maxwell’s equations [32].

$$\begin{array}{ll} & \;\left(\kappa_{a}\frac{\partial^{2}}{\partial z^{2}}\;+\;\delta \left(\left|d\right|\right)▵\kappa \frac{\partial }{\partial z}\;+\;k_{0}^{2}-\kappa_{a}n_{y}^{2}\beta_{0}^{2}\right)H_{x,m,n}\\ & \;+\kappa_{a}m_{x}n_{y}\beta_{0}^{2}H_{y,m,n} \end{array}$$

(1) $$\begin{array}{ll} & =\underset{m\prime \neq m,n^{\prime }\neq n}{\sum }\;\kappa_{\begin{array}{c} m-m^{\prime },\\ {\;}_{n-n\prime } \end{array}}\left[\left(-\frac{\partial^{2}}{\partial z^{2}}\;+\;\delta \left(\left|d\right|\right)\frac{\partial }{\partial z}\right.\right.\\ & \;\left.\left.+n_{y}n_{y}^{\prime }\beta_{0}^{2}\right)H_{x,m^{\prime },n^{\prime }}-m_{x}^{\prime }n_{y}\beta_{0}^{2}H_{y,m^{\prime },n^{\prime }}\right] \end{array}$$

Here, $\beta_{0}\;=\;\frac{2\pi }{a}$ , *m* and *n* are the wave orders in the *xy* plane, and m’ and n’ denote the source wave orders [33]. By the Fourier transform, $\frac{1}{\varepsilon \left(r\right)}$ is expanded into $\frac{1}{\varepsilon \left(r\right)}\;=\;\kappa_{a}\;+\;\sum \kappa_{\text{mn}}e^{-\text{im}\beta_{0}x-\;\text{in}\beta_{0}y}$ and $\frac{1}{\varepsilon \left(r\right)}\;=\;\frac{1}{\varepsilon_{l}}≜\kappa_{b}$ inside and outside the PCs, respectively. The guided mode resonance depends on the guided mode (*β*) and Bloch modes ( $\beta_{\text{mn}}\;=\;m\beta_{0}\hat{x}\;+\;n\beta_{0}\hat{y}$ ) at different orders (m, n), where $\beta \;=\;\left|\beta_{\text{mn}}\right|$ . Owing to geometrical symmetry, all coupling coefficients (*κ*_mn_) are symmetric at the point *Γ* = 0, resulting in complete cancelling interference. The intensity amplitudes of the in-plane and surface couplings are comparable yet opposite in sign, which effectively nullifies the total radiation (approaching zero) and consequently gives rise to stationary at-*Γ* BICs. For the accidental BICs in the *Γ* − *X* and *Γ* − *M* direction, the wave vectors form a new triangular symmetry, achieving a weighted destructive interference and the overall radiation is suppressed (close to zero). By continuously modulating the wave vectors, such weighted destructive interference always occurs with the new symmetry, resulting in tunable light trapping. Therefore, when the permittivity of the PCMs varies with the phase transition state, the out-of-plane profile of the individual channels change accordingly, modifying the coupling weights, which leads to the location shifts of the tunable BICs and the merging of the BICs. For further details and comprehensive information, please refer to the supplementary materials provided. Moreover, Δ*κ* ≜ *κb* − *κa*, representing the disparity between the coefficients of the Fourier transforms of the permittivity outside and inside the PCs, while $\delta \left(\left|d\right|\right)≜\delta \left(z-d\right)-\delta \left(z+d\right)$ denotes the surface coupling strength in TM-like mode. It is clear that $\delta \left(\left|d\right|\right)$ and ▵ *κ* contribute to the resonant modes both inside and outside the PCs. However, $\delta \left(\left|d\right|\right)$ is so weak that it barely influences the electric field of eigenmodes in the out-of-plane direction, given the extremely thin (10 nm) thickness of PCMs film [31].

**Figure 4:**
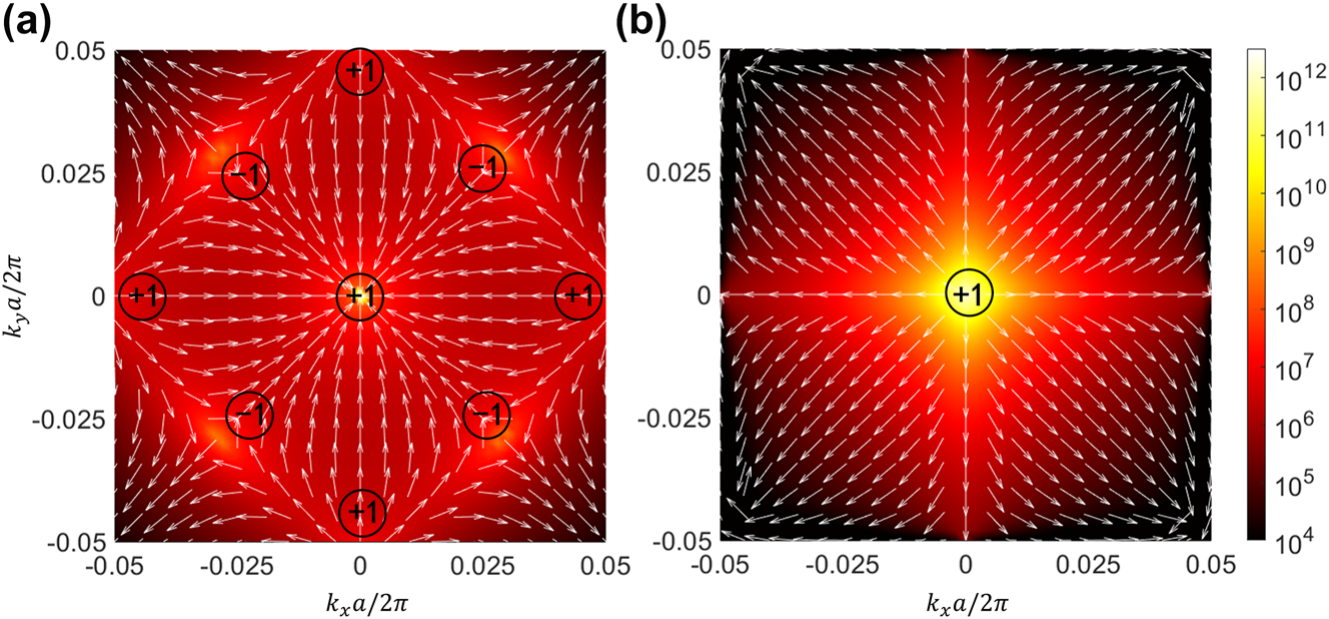
Merging multiple BICs by manipulating the phase state of PCMs. Distribution of the Q-factors calculated when the PCMs is in the amorphous (a) and crystalline (b) states, the topological charge of each BICs is marked on the plot. The polarization vectors around the BICs are indicated by the white-arrows.

The Q-factor range and topological nature of the BICs are effectively illustrated through contour plots and far-field polarization maps, as shown in Figure 4, with white arrows indicating the direction of polarization. Such BICs are vortex centers in the polarization direction of the far-field radiation, carrying a conserved and quantized topological charge defined as the number of times the polarization vector encircles the BICs [13]:

(2) $$q\;=\;\frac{1}{2\pi }\oint_{c}\text{d}k\cdot \nabla_{k}\phi \left(k\right)$$

where $c\left(k\right)\;=\;c_{x}\left(k\right)\hat{x}\;+\;c_{y}\left(k\right)\hat{y}$ represents the spatially averaged projection of the electric field onto a cell at the far-field level, pointing in the direction of polarization of the resonance in the far-field, and is referred to as the ‘polarization vector’. *φ*(*k*) = arg [*c*_*x*_(*k*) + *ic*_*y*_(*k*)] denotes the angle of the polarization vector. ***C*** in the integral represents the closed-loop path around the BIC in ***k***-space along the counterclockwise direction. When Sb_2_S_3_ is in the amorphous state, the distribution of accidental BICs exhibits a rhombic pattern. The topological charge of the central BIC and those along the *x*-axes and *y*-axes is +1, while the remaining BICs have a topological charge of −1, see result in Figure 4(a). Upon transitioning Sb_2_S_3_ to its crystalline state, the isolated BICs positioned along the high-symmetry axes converge at the central ***Γ***-point, coalescing into a single merging BICs. This merging process leads to the mutual annihilation of accidental BICs with opposing topological charges of +1 and −1, yielding a merging BICs with a net topological charge of +1. Consequently, the momentum space exhibits an expanded spectrum of wave vectors endowed with elevated Q-factors, as illustrated in Figure 4(b). More detailed information can be found in the Supplementary Information. This implies that the merging BICs is robust to external perturbations and holds potential for significant advancements in directional lasing, as well as manipulation of vortex beams [34].

### 3.2 Changing the shape of voids and manufacturing defect robustness

**Figure 5:**
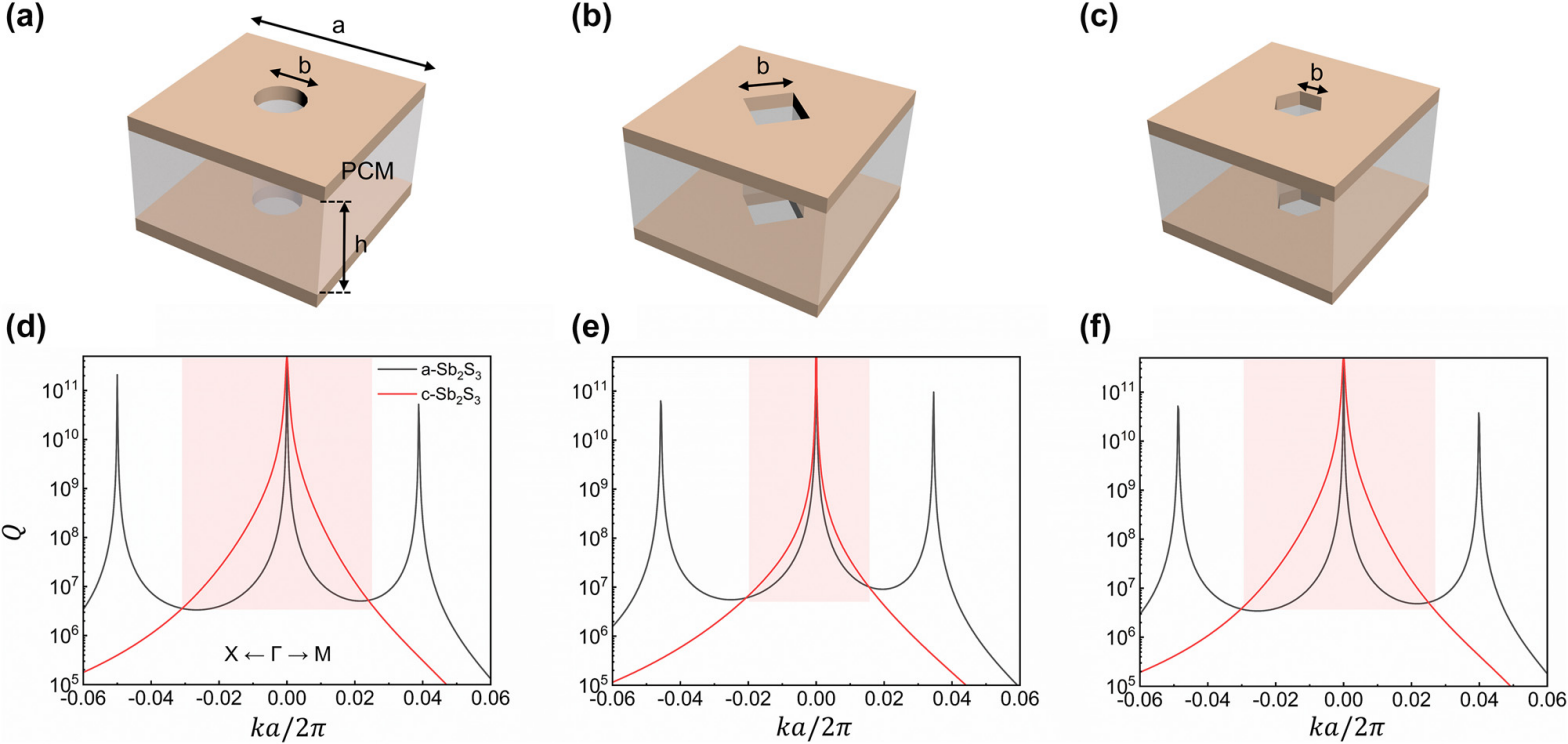
Simulated Q-factors of different unit-cell topology. The magnitude of the Q-factors (d–f) before and after the phase transition were calculated separately for square lattices with circular (a), rhombic (b) and ortho-hexagonal (c) holes. The amorphous state is indicated by the black-solid-line, the crystalline state by the red-solid-line, and the structural dimensions are labeled on the graphs.

To verify the universality of the merging BIC, we modified the geometry of the holes in the etched PCs structure, as depicted in Figure 5. Specifically, square lattices with circular (a), rhombic (b), and ortho-hexagonal (c) holes were fabricated. Despite these changes, both mirror symmetry and $C_{4}^{z}$ symmetry of the PCs remains unchanged, thereby leaving the merging of BICs unaffected. The results of variations in Q-factor of the merging BICs under these three different hole shapes are presented in Figure 5(d–f), with parameters *h* = 670 nm, 655 nm, and 685 nm while keeping other variables unchanged. One can see that accidental BICs are still able to form merging BICs as the phase state of Sb_2_S_3_ changes, irrespective of the morphology of the holes.

**Figure 6:**
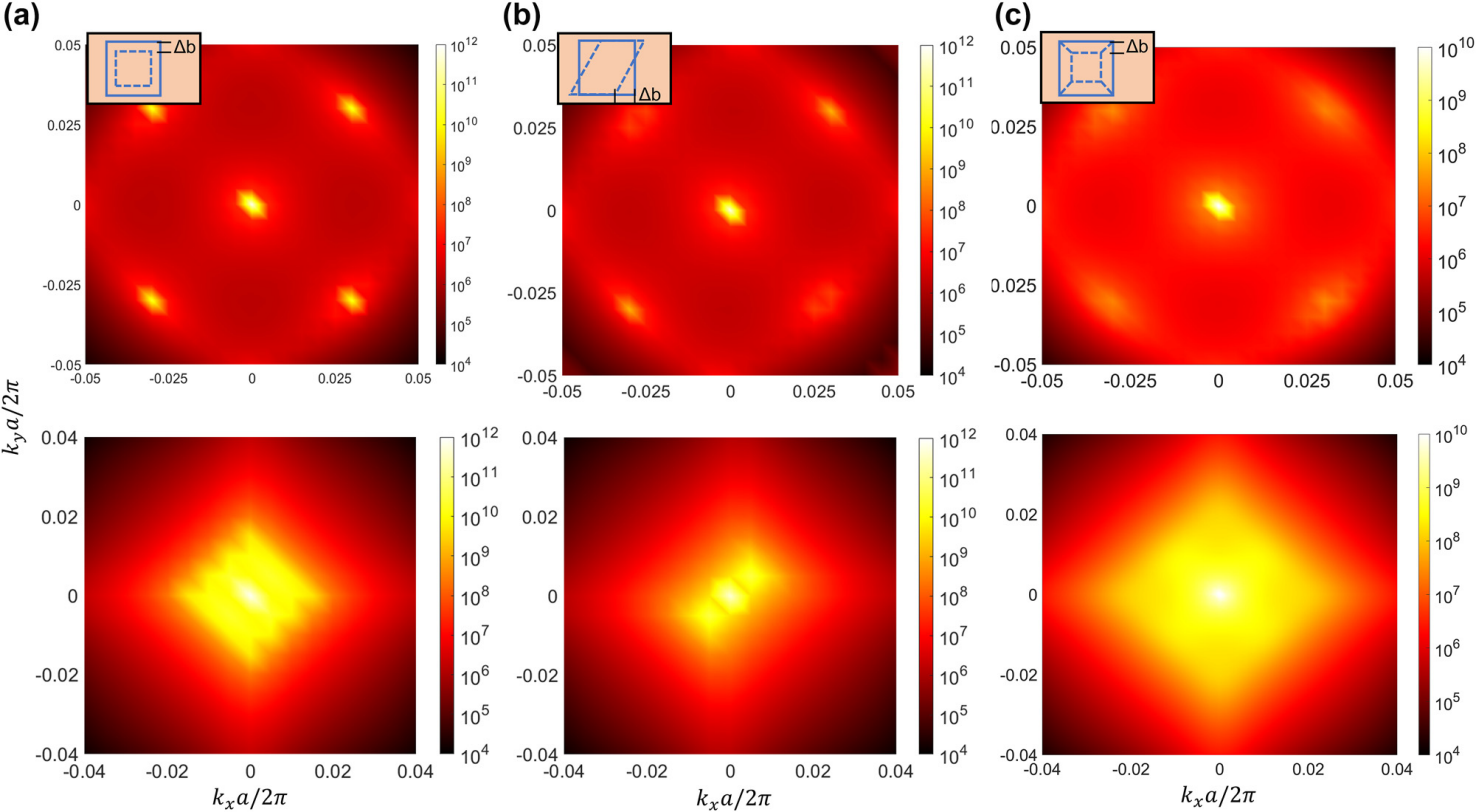
Robustness of the designed structures under different manufacturing errors. The Q-factor values for the phase transition from amorphous (first-row) to crystalline (second-row) are calculated under various conditions: when the length of the etched holes deviates from the design value (a), when the etched holes are deformed into parallelogram shapes (b), and for prismatic concave holes with different lengths at the top and bottom (c). The schematic representation of a square hole shape is shown in the upper left corner, where Δ*b* represents the degree of length change, which is 2.5 nm, 6.4 nm, and 5 nm, respectively.

Based on the results in Figure 5, it is evident that the merging BIC has the capability to achieve a higher Q-factor across a broader range of wave vectors. This characteristic enables the suppression of out-of-plane scattering losses resulting from inevitable fabrication defects. Here, we examine the stability of this structure against three types of manufacturing errors. (1) The first scenario involves a deviation in the length of the etched holes from the design value, as shown in Figure 6(a). In this instance, the symmetry remains intact and the merging case continues to exists. (2) The second scenario arises when the originally designed square holes become distorted into parallelogram-shaped apertures (Figure 6(b)), yet this distortion does not compromise the merging of BICs. (3) Subsequently, we examined a case where the square holes are substituted with prismatic-conical concave surfaces featuring varying lengths at their top and bottom ends (Figure 6(c)). In this instance, the mirror symmetry is disrupted, rendering it unfeasible to simultaneously suppress radiation in both vertical directions, consequently leading to a decrease in the Q-factor [35]. However, in all three cases, the Q-factor values of the merging BICs exhibit a significantly larger magnitude compared to those of the accidental BICs across a broader range of wave vectors. Consequently, the merging BICs demonstrate an ability to maintain a high Q-factor in the presence of manufacturing defects.

The thickness of the PCMs layer as well as the thickness difference also have an important influence on the merging BICs. By calculating the change of Q value in *k*-space under different thicknesses (8 nm–14 nm), it is found that the off-*Γ* BICs gradually move toward the *Γ* point with the increase of thickness, and complete the merger at *t* = 10 nm, producing the maximum Q values. When *t* is further increased, the merging BICs gradually undergo annihilation. This variation implies that the merging BICs exhibits a significant degree of robustness against variations in the thickness of PCMs layer. On the other hand, when the thickness difference of PCMs is Δ*t*=1 nm, due to the destruction of the mirror symmetry of the structure, only a symmetry-protected BICs at the *Γ* point is maintained. The accidental BICs degenerate into a quasi-BICs. When PCMs changes from amorphous to crystalline, the quasi-BICs move towards the *Γ* point. This finding suggests that the breaking of the symmetry induced by the thickness differences can precipitate the emergence of accidental BICs, thereby influencing the formation of merging BICs (additional details and comprehensive information can be found in the supplementary materials).

### 3.3 Inversion of topological charge

**Figure 7:**
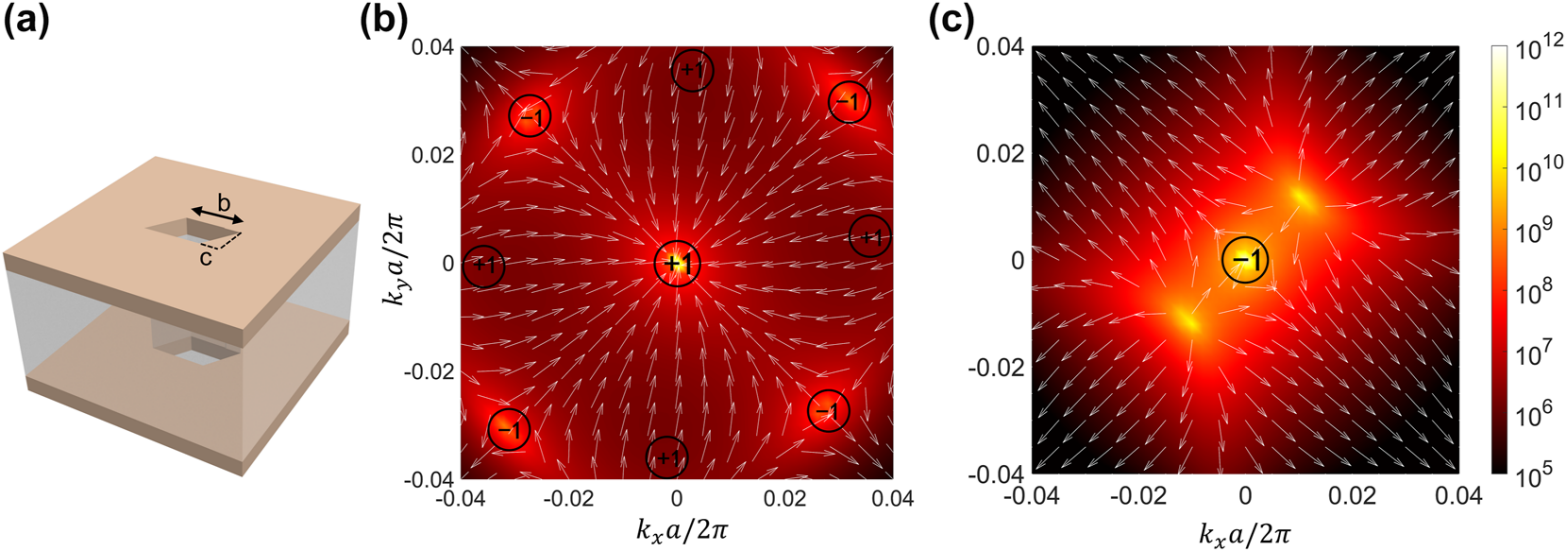
Calculated Q-factor values and polarization vectors for severe topology (square) distorted parallelogram hole. (a) Schematic diagram of the designed structure, where *c* = 0.1*b*. The Q-factor values are presented for the amorphous (b) and crystalline (c) state of the PCM, respectively, with the topological charge of the BICs marked on the plot. The white arrows denote the polarization vectors of the BICs.

When the square holes of each cell were further twisted along the *x*-axis to become parallelogram holes with increased distortion, as shown in Figure 7(a), the period and thickness remained consistent at 600 nm and 640 nm, respectively, while maintaining the PCMs thickness of 10 nm. At this point, the in-plane symmetry was reduced from $C_{2}^{z}$ to $C_{4}^{z}$ . All incidental BICs in the momentum space exhibited clockwise shifts compared to those in the square-hole case (Figure 7(b)). In this case, the BICs exhibit distinct displacements along various high symmetry axes, resulting in an asymmetric merging process. When changing the phase state of the PCMs, certain BICs initially merge in momentum space along the diagonal direction, ultimately giving rise to the emergence of a novel singular BICs at the ***Γ***-point with its topological charge reversed from −1 to +1. Therefore, despite the nonlinearity of the polarization state, it leads to the rotation of radiated light around the polarization direction of the BICs in momentum space [36]. Additional information can be found in the Supplementary information. Furthermore, in momentum space, the intrinsically entangled topology surrounding the BICs gives rise to an additional PB (Pancharatnam-Berry) phase that enables the emission of vortex light. The reversal of the topological charge holds significant potential for applications aimed at manipulating the polarization state of vortex beams near the ***Γ***-point [37], [38]. More detailed information can be found in Supplementary Information.

## 4 Conclusions

In this study, we developed an all-dielectric metasurface that integrates PCMs into PCs and verified the manipulation of BICs by phase transition. Multiple isolated BICs in the momentum space become a single merging BIC when the PCMs transforms from amorphous to crystalline state, resulting in remarkable improved Q-factor and robustness across an extended momentum region. Furthermore, we have modified the shape of the metasurface square hole to demonstrate the universality and robustness of the merging BIC against general fabrication defects. Additionally, by introducing asymmetry through twisting of the square holes, asymmetric merging is achieved along with inversion of ***Γ***-point topological charge. This control over metasurface using phase-transition states eliminates the need for adjustments in structural scaling and parameters. It significantly enhances tunability, enabling the achievement of reconfigurable polarization distributions within the radiation field. Furthermore, it holds promise for integration into photonic devices, with potential applications in optical vortices and nano-lasers.

## Supplementary Material

Supplementary Material Details
