# Supplementary material for: Dynamically tunable robust ultrahigh-Q merging bound states in the continuum in phase-change materials metasurface
Source: Nanophotonics. 2025 Jan 31;14(3):343–51. doi: 10.1515/nanoph-2024-0557 (PMC11831385; doi:10.1515/nanoph-2024-0557)
Supplement: Supplementary file 1 — Supplementary Material Details [file j_nanoph-2024-0557_suppl_001.docx]

**Supplementary Information for**

**Dynamically Tunable Robust Ultrahigh-Q Merging Bound States in the Continuum in Phase-Change Materials Metasurface**

**Hui ren^1^,** **Jietao Liu^2,*^ Zengxuan Jiang^1^, Lingyun Zhuang^1^, Botao Jiang^1^, Chunhao Xu^1^, Bo Cheng^1^and Guofeng Song^1, *^**

^1^ Institute of Semiconductors, Chinese Academy of Sciences, Beijing, China.

^2^ Institute of Intelligent Photonics, Nankai University, Tianjin, China.

*Corresponding authors: *liujietao@nankai.edu.cn;* [*sgf@semi.ac.cn*](mailto:sgf@semi.ac.cn)*;*

**This file includes:**

**Ⅰ. Optical properties of the two different PCMs**

**Ⅱ. CMT theory in photonic crystal slabs**

**Ⅲ. Manipulation of topological charge by PCMs**

**Ⅳ. The impact of varying thickness in single-layer Phase Change Materials (PCMs) and the difference in performance between single and dual-layer PCM configurations.**

**Ⅴ. The inversion of the topological charge**

**Ⅵ. Distribution of BICs in momentum space in parallelogram construction**

**Ⅶ. The proposed fabrication flow**

**Ⅰ. Optical properties of the two different PCMs**

The performance of metasurface critically depends on the optical properties of the crystalline and amorphous phases of the PCMs. **Fig.S1(a)** and **(b)** show the optical constants for the two states (amorphous and crystalline) of two different PCMs of GST and Sb_2_S_3_, respectively. It can be clearly seen that in the near infrared region, the real part of the refractive index of both PCMs is large, but the imaginary part of the refractive index of Sb_2_S_3_ is almost zero, which is much lower than that of GST. The optical loss of Sb_2_S_3_ material is much smaller than that of GST, contributes to the promoted Q-factor values of the structure exhibiting BICs resonances. To achieve higher Q value, low-loss Sb2S3 as the PCMs to modulate the resonance of the BICs is introduced and investigated.


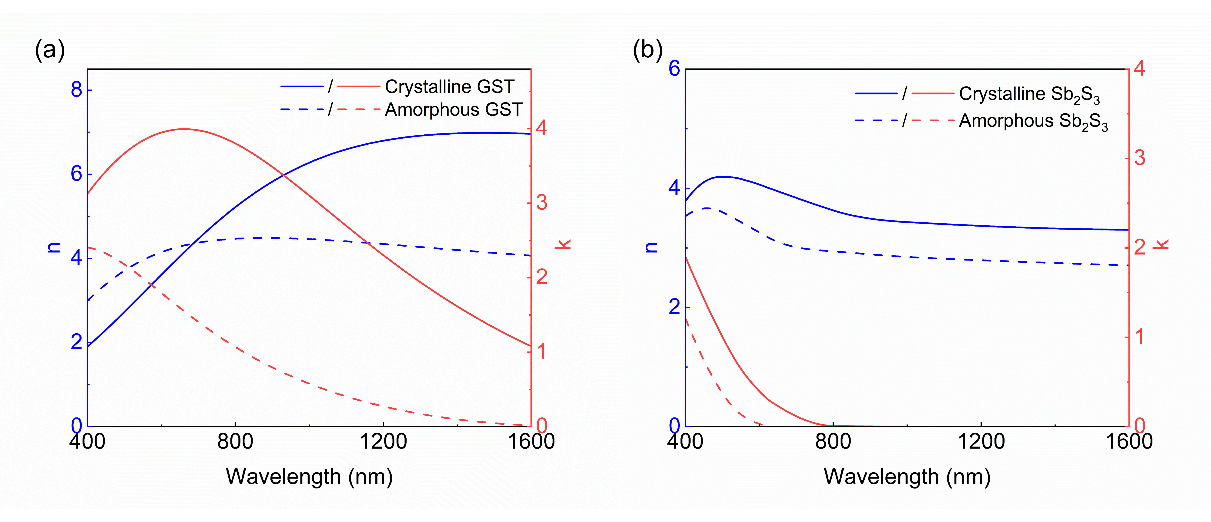


**Figure S1. Optical properties of the crystalline (solid line) and amorphous (dashed line) phase of different PCMs: (a)** GST ^[S1]^ and **(b)** Sb_2_S_3_ ^[S2]^. The blue and red curves represent the real and imaginary parts of the refractive index, respectively.

**Ⅱ. CMT theory in photonic crystal slabs**

The guided mode resonances in PCs systems interfere and couple with each other through Bloch waves (closed channels) or leaky waves (open channels), culminating in the formation of BICs. This mechanism is a fundamental aspect of how BICs are generated within these systems, as described by the coupled-mode theory (CMT) which provides a comprehensive analytical perspective on the behavior of PCs modes in the continuum. We apply the CMT theory to explain the formation of the BICs in the PCs ^[S3]^. The coupling equation, shown in **Eq.1**, is used to describe the propagation and coupling of the electromagnetic field in the PCs, including the interactions between different Bloch modes in the TM-like mode and the combined effect of in-plane and surface coupling, which explains the mechanism of the formation of the BICs. The in-plane coupling term describes the propagation of the electromagnetic field inside the PCs, and the surface coupling term describes the coupling on the upper and lower surfaces of the PCs. The guided mode resonance depends on the guided mode ($\beta$) and Bloch modes ($\beta_{mn} = m\beta_{0}\hat{x} + n\beta_{0}\hat{y}$) at different orders (m, n), where $\beta= |\beta_{mn}|$.

As shown in **Fig.S2(a)** below, the radiative wave includes the contributions from closed channels with different weights and phases. Owing to geometrical symmetry, all coupling coefficients ($\kappa_{mn}$) exhibit symmetry at the $\Gamma= 0$point, resulting in complete cancelling interference. As depicted in **Fig.S2(b)**, the intensity amplitudes of in-plane and surface couplings are comparable yet opposite in sign, which effectively nullifies the total radiation (approaching zero) and consequently gives rise to stationary at-*Γ* BICs.

For the accidental BICs at $k \simeq0.055$ in the $\Gamma-X$ direction, the wave vectors of Sx1, Rx2, and Sy2 undergo a simple merger via triangular symmetry to form a new phase matching as in **Fig.S2(c)**, and the weighted destructive interference leads to the suppression of the overall radiation (close to 0). Similarly, for another BICs occurring at $k \simeq0.04$ in the $\Gamma-M$ direction, as in **Fig.2(d)**, the wave vectors of Sx1, Sy1, and Rx2 form a new triangular symmetry, achieving a weighted destructive interference and the overall radiation is suppressed (close to 0). By continuously modulating the wave vectors, such weighted destructive interference always occurs with the new symmetry, resulting in tunable light trapping. Provided that mirror-flip and inversion symmetry are maintained, tunable BICs can be reliably identified ^[S4]^. Moreover, when the permittivity of the PCMs varies with the phase transition state, the out-of-plane profile of the individual channels change accordingly, modifying the coupling weights, which leads to the location shifts of the tunable BICs and the merging of the BICs.


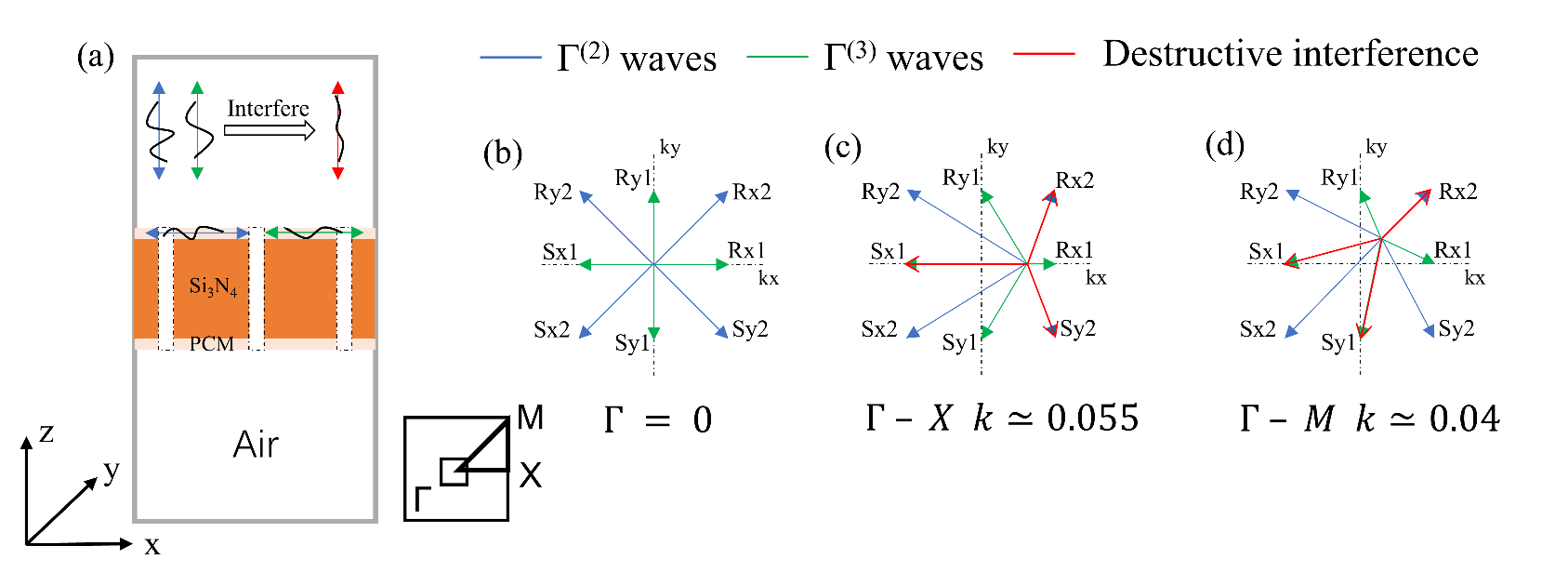


**Fig.S2 (a)** Structure: Si_3_N_4_ PCs and PCM cladding. Setting in an infinite thick layer of air above and below to ensure mirror-flip symmetry ^[S5]^. (b-d) Phase matching conditions: **(b)** $\Gamma$-point; **(c)** $k \cong0.055$ in the $\Gamma- X$ direction; **(d)** $k \cong0.04$ in the $\Gamma- M$ direction. Red arrows indicate extra degenerate wavevectors. For square lattice, the Γ^(1)^, Γ^(2)^, and Γ^(3)^ points are defined as wavevectors with lengths of 0, $2\pi/a$, and $2\sqrt{2} \pi/a$, respectively ^[S6]^.

**Ⅲ. Manipulation of topological charge by PCMs**

Using the Bloch theorem for PCs ^[S7]^, the resonant electric field can be expressed as $\boldsymbol{E}_{\boldsymbol{k}}\left( \boldsymbol{\rho},z \right)= e^{i\boldsymbol{k}\cdot\boldsymbol{\rho}}\boldsymbol{u}_{\boldsymbol{k}}(\boldsymbol{\rho},z)$, where $\boldsymbol{k} = k_{x}\hat{x}+ k_{y}\hat{y}$ is the two-dimensional wave vector, $\boldsymbol{\rho}= x\hat{x}+ y\hat{y}$ are the plane coordinates, $\boldsymbol{u}_{\boldsymbol{k}}$ is the periodic function of $\boldsymbol{\rho}$, and $z$ is the direction normal to the PCs. The resonance amplitude can be expressed as the zero-order Fourier coefficients of $\boldsymbol{u}_{\boldsymbol{k}}$ as $\boldsymbol{c}\left( \boldsymbol{k} \right)= c_{x}\left( \boldsymbol{k} \right)\hat{x} + c_{y}\left( \boldsymbol{k} \right)\hat{y}$, where $c_{x}\left( \boldsymbol{k} \right)= \hat{x} \cdot\left\langle\boldsymbol{u}_{\boldsymbol{k}} \right\rangle$ and $c_{y}\left( \boldsymbol{k} \right)= \hat{y} \cdot\left\langle\boldsymbol{u}_{\boldsymbol{k}} \right\rangle$. The brackets indicate the spatial average of a cell in any horizontal plane outside the slab. $\boldsymbol{c}\left( \boldsymbol{k} \right)$ is the projection of $\left\langle\boldsymbol{u}_{\boldsymbol{k}} \right\rangle$ onto the $x - y$ plane, pointing in the direction of the polarization of the far-field resonance, and hence $\boldsymbol{c}\left( \boldsymbol{k} \right)$ is called the ‘polarization vector’. When $c_{x}= c_{y}=0$, the outgoing power is zero and the resonance is transformed into BICs, which appears on the nodal lines of $c_{x}$ and $c_{y}$ ^[S8]^. Such a nodal intersection causes a vortex in the polarization vector field centered on the BICs, exhibiting the feature of the topological charge $q$ of the BICs, that's **Eq. 2** in the main text. The winding number of the polarization vector along a closed path is given by the sum of the topological charges carried by all BICs enclosed within this path. When keeping the symmetry of the system, the number of windings defined on this path remains constant, which means that the topological charge is a conserved quantity.

When PCMs is in the amorphous state, the symmetry-protected BICs located at the $\Gamma$-point and the accidental BICs at the eight high symmetry points are calculated, and the direction of their polarization vector fields is shown in the **Fig.S3(a)**. When the PCM transitions to the crystalline state, the PCs cladding permittivity changes, resulting in the location shifts of the tunable BICs towards the $\Gamma$-point. Accidental BICs with opposite charges meet at the $\Gamma$-point, and according to the principle of charge conservation, these BICs annihilate, leaving merging BICs with charge of +1 (**Fig.S3(b)**). It is worth pointing out that if two BICs with the same topological charge meet at the $\Gamma$-point, they will not annihilate and will only be deflected towards the $k_{y}$ axis ^[S7]^.


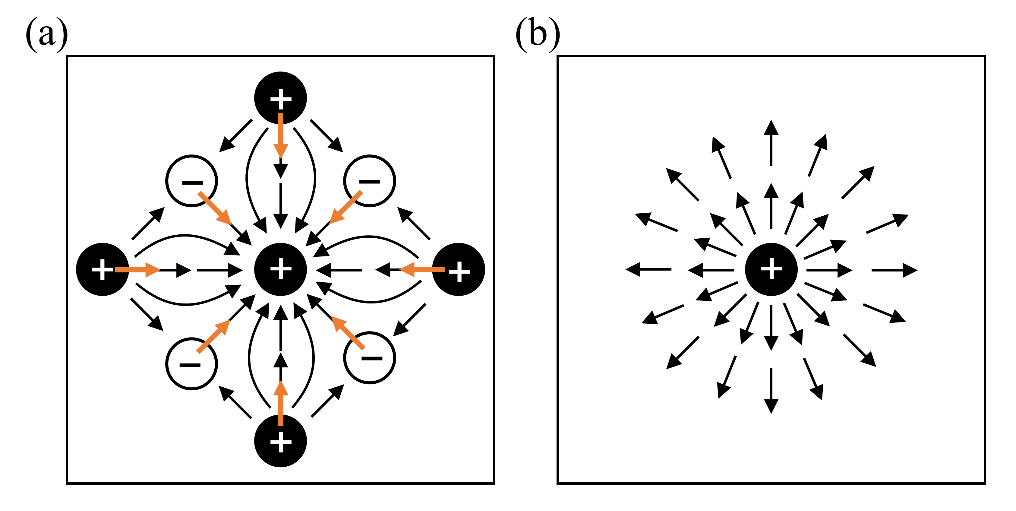


**Fig.S3** Directions of the polarization vector field before **(a)** and after **(b)** the phase transition of PCMs showing vortices with a topological charge of ±1 at each $k$ points. The orange arrows indicate the direction of movement of the BICs.

**Ⅳ. The impact of varying thickness in single-layer Phase Change Materials (PCMs) and the difference in performance between single and dual-layer PCMs configurations.**

The thickness of the PCMs layer as well as the thickness difference indeed play an important role in influencing the merging BICs, and changes in the structural parameters can cause merging and annihilation of BICs ^[S5, S9]^. We complementarily calculated the variation of Q values in *k* -space when the thickness ($t$) of the PCMs layer is 8 nm, 9 nm, 10 nm, 11 nm, 12 nm, and 14 nm, the results are shown in the **Fig. S4**. One can see that as the thickness increases, the off-$\Gamma$ BICs gradually move toward the $\Gamma$ point with the phase transition and complete the merger at $t = 10 nm$, producing the maximum Q values. When $t$ is further increased, the merging BICs gradually undergo annihilation. By examining the variation of the Q values, it has been observed that the merging BICs exhibits a significant degree of robustness against variations in the thickness of PCMs layer. This robustness is a crucial characteristic for practical applications, as it implies that the performance of the metasurface remains stable despite minor fluctuations in the PCM layer thickness.


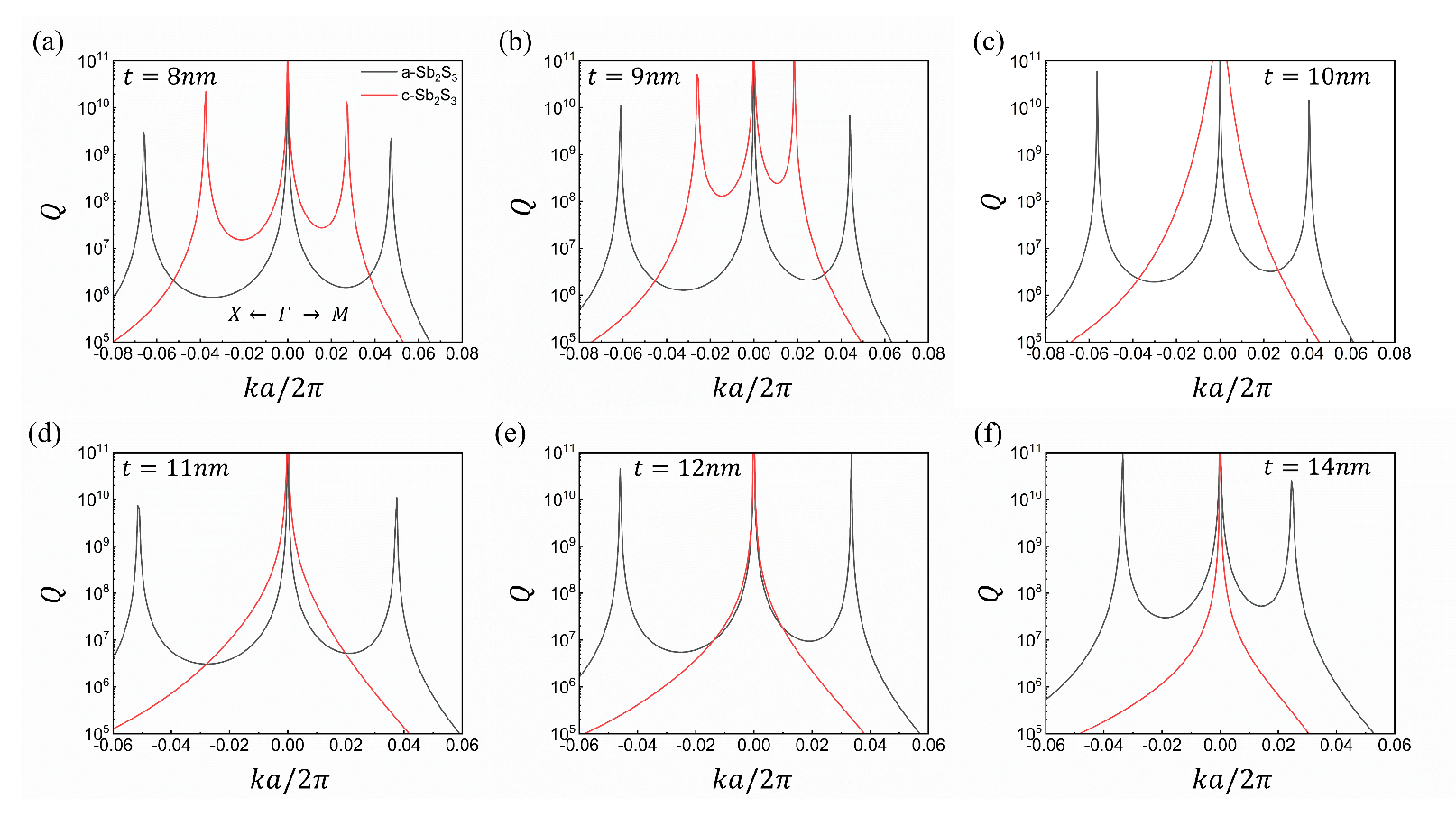


**Fig.S4** The variation of the Q values of the metasurface for different PCMs layer thicknesses.

On the other hand, when we set the thickness difference of the PCMs layer to $\Delta t=1nm$, see result in the **Fig.S5**, due to the destruction of the mirror symmetry of the structure, only a symmetry-protected BICs at the$\Gamma$ point is maintained. The accidental BICs degenerate into a quasi-BICs. When PCMs changes from amorphous to crystalline, the quasi-BICs move towards the $\Gamma$ point. This finding suggests that the thickness variation does not significantly impact the tuning of BICs. However, the breaking of the symmetry induced by the thickness differences can precipitate the emergence of accidental BICs, thereby influencing the formation of merging BICs.


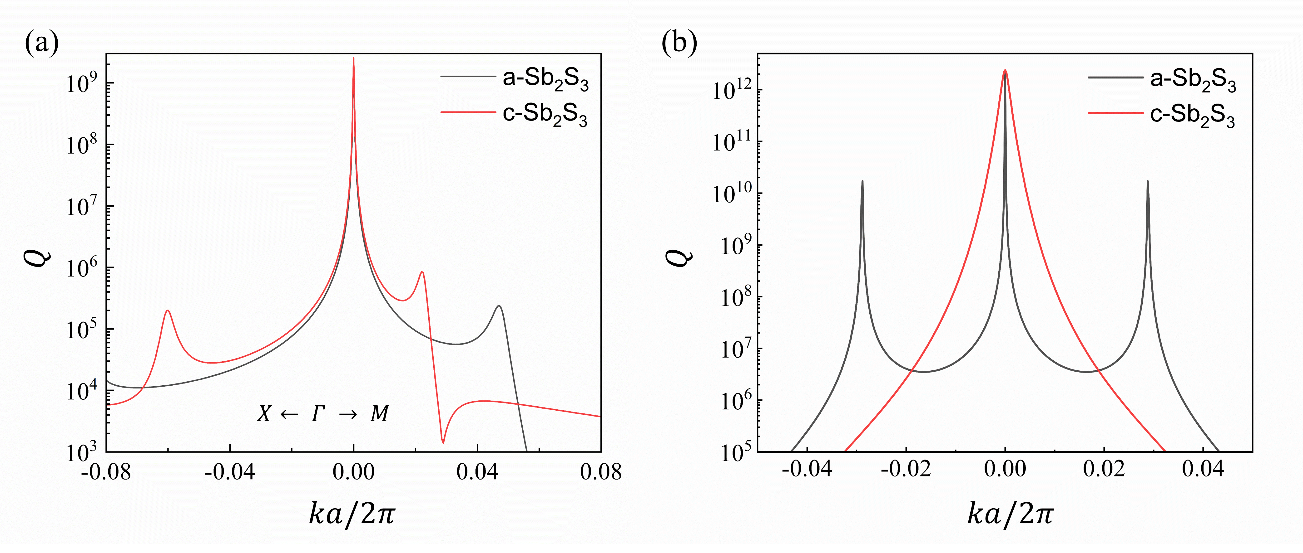


**Fig.S5** The Q-values in k-space for **(a)** PCMs thickness difference $\Delta t = 1 nm$ and **(b)** without thickness difference

**Ⅴ. The inversion of the topological charge**

As described in the manuscript, BICs exhibit topological vortex in momentum space, characterized by an integer topological charge of ±1, which is defined as **Eq. 1**.

$q=\frac{1}{2\pi}\oint_{C} dk\cdot\nabla_{k}\phi\left( k \right)$ (1)

where $\boldsymbol{c}\left( \boldsymbol{k} \right)$ denotes the spatially averaged projection of the electric field in the $x-y$ plane, pointing in the direction of polarization of the far-field resonance, which is called the “polarization vector”. As shown in **Fig.S6(a)** below, the green and red dashed lines (including the solid lines on the axes) represent the nodal lines of $c_{x}$ and $c_{y}$ in momentum space. When the outgoing power is zero, the resonance is transformed into BICs, where $c_{x}= c_{y}=0$, and the BICs occur at the intersection between the nodal lines of $c_{x}$ and $c_{y}$ ^[S8]^.

By twisting the square holes of each cell into parallelogram holes along the *x*-axis, while keeping other geometrical parameters constant, all off-$\Gamma$BICs exhibit significant displacements along various high symmetry axes. When changing the phase state of PCMs, certain BICs first merge in momentum space along the diagonal direction, eventually creating new merging BICs at$\Gamma$-point with their topological charge reversed from +1 to -1.

To illustrate more clearly the evolution of merging BICs in the parallelogram structure, we calculated the nodal lines of $c_{x}$ and $c_{y}$ as shown in **Fig.S6(b)**. In the case of square holes, both nodal lines are circular and the intersections are BICs. When the square holes are twisted into parallelogram holes, the nodal lines are stretched and squeezed in *k*-space toward $k_{x}$ and $k_{y}$, respectively. This situation changes the intersection between $c_{x}$ and $c_{y}$, thus asymmetrically adjusting these accidental BICs. In the region of $k_{x}$ < 0, the proximal semicircle of the curve $c_{x}=0$ shifts downward, whereas in the region of $k_{x}$ > 0, the symmetry circle shifts in the opposite direction. The similar situation occurs in the left and right directions of the curve $c_{y}=0$. Thus, the accidental BICs with topological charge -1 move symmetrically towards the $\Gamma$-point, while those with topological charge +1 move asymmetrically towards the $\Gamma$-point.

Therefore, BICs on different axes have different scales of movement under this structure. The negative topological charges in the original $\Gamma-M$ direction move closer to the positive BICs in the $\Gamma-X$ direction and to the center BIC of the$\Gamma$-point, and then merge with those positive topological charge BICs that have moved with greater magnitude. They eventually produce a new merging BICs with a topological charge of -1 at $\Gamma$-point. The nodal line is shown in **Fig.S6 (c)**.


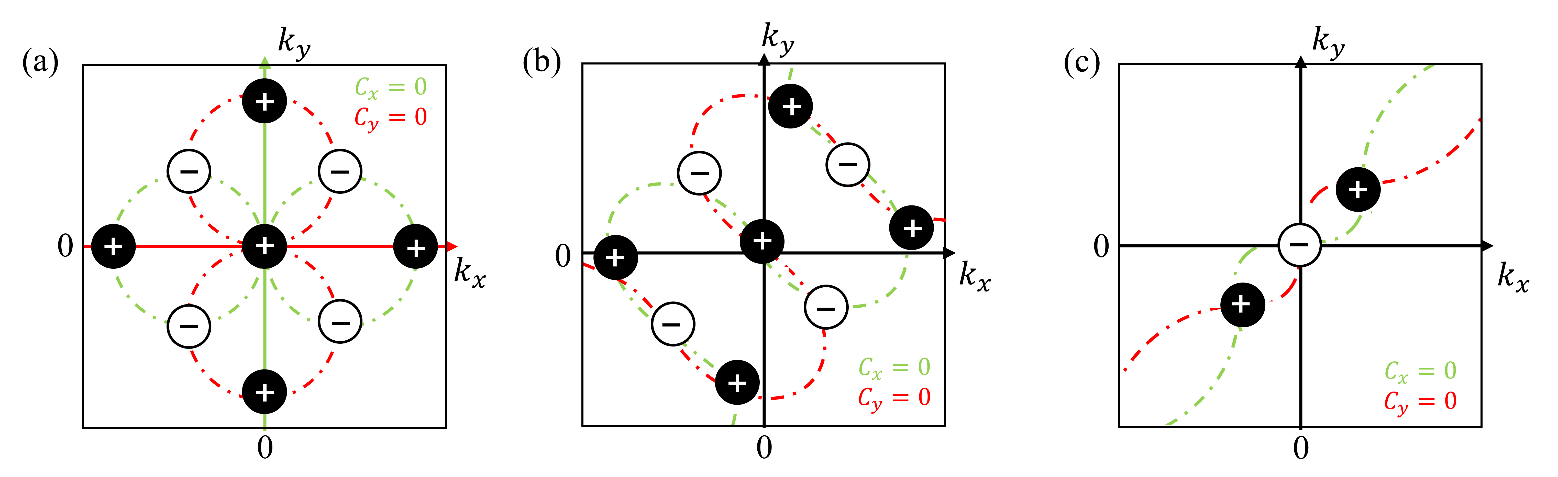


**Fig.S6** The nodal line schematics of $c_{x}$ (green) and $c_{y}$ (red) in *k*-space are presented for the square-hole shape **(a)** and parallelogram-hole shape **(b-c)**. It is worth noting that **(a-b)** are for amorphous state PCMs, while **(c)** is for PCMs in its crystalline state.

**Ⅵ. Distribution of BICs in momentum space in parallelogram construction**

When the topology of the etched holes is distorted into parallelograms, the mirror symmetry is broken while the in-plane rotational symmetry is still preserved, the accidental BICs are redistributed. To illustrate the evolution of the merging BICs in the parallelogram structure more clearly, we calculate the Q-factor values and polarization vectors of the PCs for the cases of $c = 0.025b$ and $c = 0.25b$, respectively, as shown below in **Fig. S7**. One can see that as the degree of parallelogram distortion increases, the contingent BICs in the momentum space move clockwise with greater magnitude. In this case, when changing the phase state of the PCMs, part of the BICs merge to form an isolated BICs since the BICs on different highly symmetry axes have different moving scales.


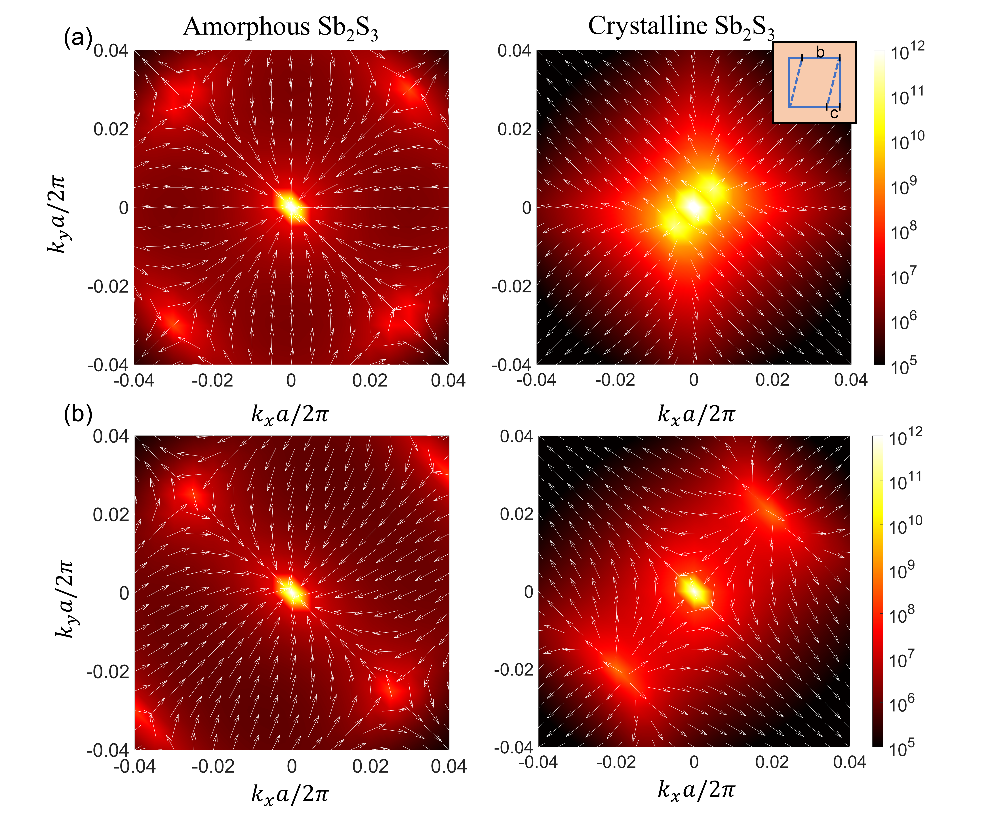


**Figure S7.** **Calculated Q-factor distribution of BICs and polarization vectors in parallelogram holes** when *c=0.025b* **(a)** and *c=0.25b* **(b)**, respectively. The Q-factor values are presented for the amorphous (left) and crystalline (right) state of the PCMs, and the white arrows denote the polarization vectors of the BICs.

**Ⅶ. The proposed fabrication flow**

The designed experimental scheme is shown in **Fig.S8(a)**. Initially, a 10 nm layer of Sb_2_S_3_ is deposited on a Si substrate via radio frequency (RF) sputtering ^[S10, S11]^. Subsequently, a 640 nm layer of Si_3_N_4_ is deposited using plasma-enhanced chemical vapor deposition (PECVD). Thereafter, an additional 10 nm layer of Sb_2_S_3_ is deposited via RF sputtering, as well. A hard-mask is formed by depositing SiO_2_ through PECVD, followed by the spin-coating of photoresist such as AZ-1500. After electron beam exposure, reactive ion etching (RIE) and inductively coupled plasma (ICP) etching are employed to pattern the hard-mask to create the square-hole structure. The photoresist and hard mask are then removed, and ultimately, the Si substrate is etched away to complete the structure. A three-dimensional cross-sectional diagram of the sample is depicted in the **Fig.S8 (b)**.


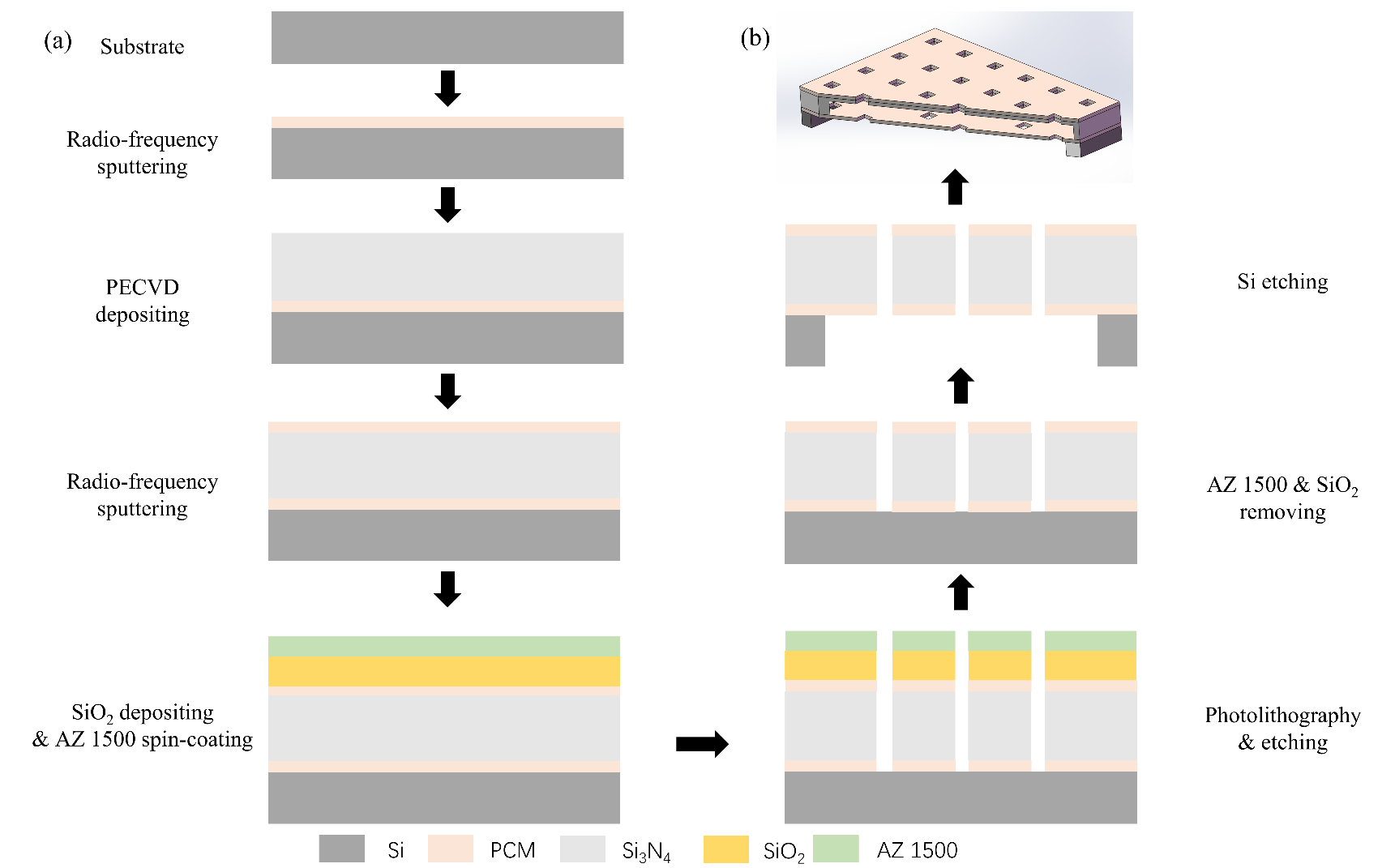


**Fig.S8** Schematic diagram of fabrication flow **(a)** and three-dimensional cross-section **(b)** of the metasurface.

**References**

[S1] A. Barreda *et al.*, "Tuning and switching effects of quasi-BIC states combining phase change materials with all-dielectric metasurfaces," *Optical Materials Express,* Article vol. 12, no. 8, pp. 3132-3142, Aug 1 2022.

[S2] M. Delaney, I. Zeimpekis, D. Lawson, D. W. Hewak, and O. L. Muskens, "A New Family of Ultralow Loss Reversible Phase-Change Materials for Photonic Integrated Circuits: Sb_2_S_3_ and Sb_2_Se_3_," *Advanced Functional Materials,* Article vol. 30, no. 36, Sep 2020, Art. no. 2002447.

[S3] Y. Yang, C. Peng, Y. Liang, Z. Li, and S. Noda, "Analytical Perspective for Bound States in the Continuum in Photonic Crystal Slabs," *Physical Review Letters,* Article vol. 113, no. 3, Jul 15 2014, Art. no. 037401.

[S4] C. W. Hsu *et al.*, "Observation of trapped light within the radiation continuum," *Nature,* Article vol. 499, no. 7457, pp. 188-191, Jul 11 2013.

[S5] G. Zhu, S. Yang, and J. C. Ndukaife, "Merging toroidal dipole bound states in the continuum without up-down symmetry in Lieb lattice metasurfaces," *Nanophotonics,* Article vol. 13, no. 9, pp. 1561-1568, Apr 22 2024.

[S6] S. Iwahashi, Y. Kurosaka, K. Sakai, K. Kitamura, N. Takayama, and S. Noda, "Higher-order vector beams produced by photonic-crystal lasers," *Optics Express,* Article vol. 19, no. 13, pp. 11963-11968, Jun 20 2011.

[S7] B. Zhen, C. W. Hsu, L. Lu, A. D. Stone, and M. Soljacic, "Topological Nature of Optical Bound States in the Continuum," *Physical Review Letters,* Article vol. 113, no. 25, Dec 18 2014, Art. no. 257401.

[S8] W. Chen, Y. Chen, and W. Liu, "Singularities and Poincare Indices of Electromagnetic Multipoles," *Physical Review Letters,* Article vol. 122, no. 15, Apr 19 2019, Art. no. 153907.

[S9] Y. Gao, J. Ge, Z. Gu, L. Xu, X. Shen, and L. Huang, "Degenerate merging BICs in resonant metasurfaces," *Optics letters,* vol. 49, no. 23, pp. 6633-6636, 2024-Dec-01 2024.

[S10] Z. Fang, J. Zheng, A. Saxena, J. Whitehead, Y. Chen, and A. Majumdar, "Non-Volatile Reconfigurable Integrated Photonics Enabled by Broadband Low-Loss Phase Change Material," *Advanced Optical Materials,* Article vol. 9, no. 9, May 2021, Art. no. 2002049.

[S11] A. Alquliah *et al.*, "Reconfigurable metasurface-based 1 x 2 waveguide switch," *Photonics Research,* Article vol. 9, no. 10, pp. 2104-2115, Oct 1 2021.
